# Multivalent display of VP28 on chimeric virus-like particles enhances binding to shrimp target tissues: A novel antiviral strategy against white spot syndrome virus

**DOI:** 10.14202/vetworld.2025.2194-2205

**Published:** 2025-08-02

**Authors:** Somkid Jaranathummakul, Pitchanee Jariyapong, Orawan Thongsum, Supawich Boonkua, Charoonroj Chotwiwatthanakun, Monsicha Somrit, Somluk Asuvapongpatana, Attaboon Wathammawut, Wattana Weerachatyanukul

**Affiliations:** 1 Department of Anatomy, Faculty of Science, Mahidol University, Bangkok, Thailand; 2 Department of Medical Science, School of Medicine, Walailak University, Thasala District, Nakhonsrithammarat, Thailand; 3 Academic and Curriculum Division, Nakhonsawan Campus, Mahidol University, Nakhonsawan, Thailand; 4 Department of Anatomy, Faculty of Medicine, Sri Nakharinwiroj University, Bangkok, Thailand

**Keywords:** aquaculture vaccines, *Macrobrachium rosenbergii* nodavirus, shrimp immunity, tissue binding, virus-like particles, viral protein 28, white spot syndrome virus

## Abstract

**Background and Aim:**

White spot syndrome virus (WSSV) is a devastating pathogen in shrimp aquaculture, with viral protein 28 (VP28) playing a critical role in host cell attachment and entry. The extracellular domain of VP28 (residues 35–95) is immunogenic and essential for infection; however, its receptor interaction mechanisms remain incompletely elucidated. This study aimed to evaluate the tissue-binding affinity of full-length VP28 and its derived peptides (P1: Residues 35–65; P2: Residues 66–95) as well as a multimeric chimeric virus-like particle (K5-VLP) displaying VP28 on the surface of *Macrobrachium rosenbergii* nodavirus capsids to enhance host tissue interaction.

**Materials and Methods:**

Recombinant VP28, synthetic peptides (P1, P2), and chimeric K5-VLP were produced and characterized. Binding and inhibition assays were performed using enzyme-linked immunosorbent assay and immunofluorescence microscopy on shrimp gill, hemocyte, muscle, stomach, and hepatopancreas tissues.

**Results:**

Full-length VP28 exhibited strong binding to gill, hemocyte, and muscle tissues. The P1 and P2 peptides showed moderate binding compared to rVP28. Notably, K5-VLP demonstrated a 1.7-fold higher binding affinity than rVP28 in gill tissues and significantly outperformed P1 and P2 peptides. Inhibition assays confirmed that K5-VLP more effectively interfered with VP28 binding than peptides. Structural analysis and transmission electron microscopy confirmed correct assembly and surface presentation of VP28 on the VLPs.

**Conclusion:**

Multimeric display of VP28 on K5-VLP enhances its binding affinity to shrimp tissues compared to monomeric or peptide forms. This suggests a promising platform for antiviral strategies, including competitive inhibition of WSSV entry and targeted therapeutic delivery in shrimp aquaculture.

## INTRODUCTION

White spot disease, caused by the white spot syndrome virus (WSSV), remains one of the most devastating and economically significant threats to the global aquaculture industry. WSSV possesses a broad host range, infecting freshwater prawns, penaeid shrimp, and a variety of other crustaceans, including lobsters and crabs [1]. This double-stranded DNA virus belongs to the Nimaviridae family and features a circular genome approximately 300 kb in length. The virus exhibits considerable genetic variation, with open reading frames ranging from 515 to 684 amino acids among different geographic isolates [2, 3]. Structurally, WSSV consists of a nucleocapsid enclosed within a trilaminar envelope, along with a distinct tail-like appendage of yet undefined function [4, 5]. Among its structural components, viral protein 28 (VP28) is the most abundant envelope-associated protein and plays a pivotal role in host interaction.

VP28 serves as a viral attachment ligand, facilitating entry into host cells by interacting with several cytosolic proteins, including PmRab7, heat shock cognate protein 7, and CS [6–10]. Despite these known interactions, the exact membrane-bound receptor responsible for mediating VP28 attachment remains unidentified. In addition, VP28 is known to stimulate the immune system by increasing hemocyte circulation and enhancing phagocytic activity in shrimp [11]. Other structural proteins, including VP26, act as tegument components, linking the viral envelope to the nucleocapsid and contributing to virion assembly [12, 13].

Recent studies employing truncated variants of VP28, specifically VP28-∆N (residues 1–27), ∆M (35–95), and ∆C (115–204), revealed that deletion of the central domain (∆M) resulted in the lowest survival rate (~20%) among infected shrimp [14]. This indicates that the 35–95 amino acid region is a critical functional domain for receptor interaction. Moreover, this segment has demonstrated potent immunogenicity and a strong capacity to inhibit viral infection.

Beyond the linear peptide format, VP28 has been reformulated into dimeric and tetrameric constructs, which exhibit enhanced antiviral efficacy against WSSV [14–16]. Building on this concept, we developed a novel multivalent VP28 platform by genetically engineering a *Macrobrachium rosenbergii* nodavirus virus-like particle (MrNV-VLP) to present the VP28 peptide on its surface. This chimeric VLP, referred to as K5-VLP, displays up to 180 copies of VP28, significantly enhancing its valency and mimicking the native viral structure. Unlike monomeric or truncated VP28, this multimeric platform was evaluated for its direct tissue-binding capability in shrimp [17]. The high-density presentation of VP28 on the K5-VLP scaffold significantly enhanced binding affinity and immunogenic pote-ntial, surpassing the performance of oligomeric VP28 forms [16].

WSSV remains a significant threat to global shrimp aquaculture, with no effective antiviral treatments or commercial vaccines currently available. While the envelope protein VP28 has been extensively studied for its critical role in mediating viral entry and inducing immune responses in shrimp, its interaction with host cell receptors remains incompletely characterized. Previous studies have demonstrated that truncated and multimeric forms of VP28 can offer partial protection and elicit immune responses; however, these formats still suffer from limited binding efficiency and inconsistent protection across tissue types. Moreover, although VLPs derived from MrNV have shown promise as immunogenic delivery platforms, the potential of multimeric display of VP28 peptides on such VLPs to improve tissue targeting and receptor binding has not been thoroughly investigated. There is a critical knowledge gap in understanding how multivalent presentation of functional VP28 domains affects tissue-level binding, immune engagement, and interference with native virus attachment–factors essential for developing next-generation antiviral strategies.

This study aims to develop and evaluate a novel chimeric VLP (K5-VLP) presenting multimeric VP28 peptides on the surface of MrNV capsid proteins. Specifically, we aim to (1) engineer and characterize the structural integrity of K5-VLPs with surface-displayed VP28-derived peptides, (2) compare the tissue-binding affinity of full-length VP28, its derivative peptides (P1 and P2), and multimeric K5-VLP in key shrimp tissues including gills and hemocytes, and (3) assess the competitive inhibition potential of K5-VLP against native VP28-host interactions. Through this approach, we aim to determine whether the multivalent display of VP28 on VLPs provides a structural and functional advantage that can be harnessed for targeted therapeutic delivery or vaccine development in shrimp aquaculture.

## MATERIALS AND METHODS

### Ethical approval

Animal handling, shrimp tissue collection, and hemocyte culture were performed according to the guidelines of the Animal Care Committee of the Faculty of Science, Mahidol University (protocol # MUSC65-027-620).

### Study period and location

The study was conducted from January 2022 to December 2024 at the Department of Anatomy, Faculty of Science, Mahidol University.

### Peptide design and synthesis

Two short peptides of VP28, including P1 (residues 35–65) and P2 (residues 66–95), were selected based on their extracellular localization and putative role as WSSV-binding domains [14]. As shown in Table 1, the amino acid sequence of P1 spans from Lys35 to Phe65 (Molecular weight [MW] = 3690.09 g/mol), and that of P2 extends from Lys66 to Asp95 (MW = 3614.06 g/mol). The control peptide was synthesized using six tandem repeats of the Glycine/Alanine/threonine tri-amino acid, resulting in an 18-amino-acid control sequence (MW = 1620 g/mol). All peptides were biotinylated at the N-terminus and synthesized with ≥96% purity (Ward Medic Ltd, Bangkok, Thailand). The obtained peptide powders were dissolved in 100% dimethyl sulfoxide (DMSO) as indicated by the manufacturer at a final concentration of 1 mg/mL. The chimeric plasmid expressing MrNV-VLP fused with VP28 (35–65), hereafter referred to as K5-VLP, was constructed using the MrNV capsid (GenBank: EU150129) and WSSV VP28 (GenBank: AKS25343.1). In general, the vector of chimeric K5-VLP contained 1113 bp of the entire MrNV-VLP capsid gene sequence, 45 bp of a flexible amino acid linker (GGGGSGGGGSGGGGS), 93 bp of VP28, 18 bp of a hexa-histidine tag, and 3 bp of a stop codon. Structural predictions for the chimeric K5-VLP protein were generated using the Phyre2 program (Structural Bioinformatics Group, Imperial College, London, England - http://www.sbg.bio.ic.ac.uk) and visualized using UCSF Chimera (Computational Graphics Lab, University of California, San Francisco, California, USA - http://www.cgl.ucsf.edu).

**Table 1:** VP28 peptide (P1 and P2) and control (mock) peptide sequences.

Peptides	Sequence
VP28_(35-65)_ (P1)	KTIETHTDNIETNMDPNLRIPVTAEVGSGYF
VP28_(66-95)_ (P2)	KMTDVSFDSDTLGKIKIRNGKSDAQMKEED
Control (mock) peptide (M)	GATGATGATGATGATGAT

VP28=Viral protein 28

### Construction and expression of VLP

Plasmid construction, transformation, protein expression, and purification were performed according to previously established protocols by Jariyapong *et al*. [18]. The K5-VLP construct was ligated into the plasmid for the expression by T7 RNA polymerase-24 or pET-24(+) between BamHI and HindIII restriction sites and transformed into BL21-strained *Escherichia coli*. Positive bacterial clones were selected and incubated in Luria Broth (LB) medium containing 10 μg/mL of kanamycin and incubated at 37°C overnight. Bacterial cells were further incubated in 250 mL LB medium containing 10 μg/mL kanamycin at 37°C until the 600-nm absorbance reached 0.6–0.8. Protein expression was induced by the addition of 1 mM isopropyl-beta-D-thiogalactoside at 20°C for 18 h. Bacterial pellets were collected by centrifugation at 6000× *g* for 5 min at 4°C and then lysed using 1% sarcosyl in phosphate-buffered saline (PBS, pH 7.4), followed by reciprocal sonication at 20 Hz and 100 W for 5 min. After centrifugation to discard cell debris, soluble proteins in the supernatant were purified using a nickel nitrilotriacetic acid (NTA) affinity chromatography. Desalting was performed using a centrifugal concentrator with a 10 kDa MW cutoff. A NanoDrop 2000 spectrophotometer was used at 280 nm (Thermo Fisher Scientific, Waltham, MA) for quantification.

### Protein profiling and antibody specification

Proteins from the purification step, including wholecell lysates, flow-through, eluents, and reten-tates, were resolved by 10% sodium dodecyl sulfate-polyacrylamide gel electrophoresis and stained with Coomassie Brilliant Blue R-250. The resolved proteins were transferred to polyvinylidene fluoride (PVDF) membranes (Millipore, Billerica, MA) for Western blot analysis. Membranes were blocked with 2% BSA and 5% skim milk in PBS to prevent non-specific binding at room temperature for 2 h, followed by exposure to 1:2,000 anti-VP28 polyclonal antibody (Abcam, Cambridge, UK, cat# ab26935), anti-His (Abcam, cat# ab18184), or anti-MrNV monoclonal antibody (a kind gift from Prof. Dr. Paisan Sithigorngul, Srinakarinwiroj University, Bangkok, Thailand), which has been shown to confer high specificity against MrNV capsid protein [19]. The membrane was further exposed to 1:5,000 goat anti-mouse immunoglobulin G (IgG) secondary antibody conjugated with horseradish peroxidase (HRP) (Abcam, cat# ab97023). An Immobilon Crescendo Western HRP substrate was used to detect the antibody-antigen complex (Amersham Biosciences, Piscataway, NJ, cat# WBLUR0500).

### Structural validation

For VLP structure verification by transmission electron microscopy (TEM), 10 μL of purified chimeric K5-VLP was pipetted onto 200-mesh formvar-carbon-coated electron microscopy (EM) grids (Electron Microscopy Sciences, Hatfield, PA, USA) and allowed to settle on the grid surface for 30 s. Non-adhering VLPs were blotted away using filter paper, and the adhered ones on the TEM grid were washed multiple times by dipping them into Milli-Q water breakers. A 2% uranyl acetate solution was applied to the VLP-coated grids for 30 s, followed by filter paper blotting. After letting the samples dry for 2 h, they were viewed under 50,000× magnification using a transmission electron microscope operated at 120 kV (JEOL 1230, Tokyo, Japan).

### Functional assays

#### Enzyme-linked immunosorbent assay (ELISA)

Shrimp tissues were carefully collected from the gill, muscle, stomach, hepatopancreas, and hemocytes. Whole tissue proteins were extracted and coated onto 96-well microtiter plates at 4°C overnight using a coating buffer (0.1 M Na_2_CO_3_, 0.1 M NaHCO_3_, pH 9.5). The coated proteins were washed twice with PBS containing 0.2% Tween-20 (PBST). Non-specific binding was blocked with 5% BSA in PBST for 1 h at room temperature. Initially, standard curves of ELISA in the individual tissues were generated with 0.31–5.0 μg in 50 μL of VP28 to obtain a clue for the proper VP28 treatment. The tissues were then incubated overnight at 4°C with 2.5 and 5.0 μg (equivalent to 0.045 and 0.089 nmol) of biotinylated-VP28 in 50 μL. In addition, the coated proteins were treated separately with 2.5 μg of biotinylated-P1 or -P2 peptide (0.64 nmol), biotinylated-control mock (M) peptide (1.28 nmol), or chimeric K5-VLP or parental MrNV-VLP (VLP control) (0.000275 nmol) in 50 μL of 0.02% DMSO and blank control (0.02% DMSO) at 4°C overnight. The same concentrations of VP28, P1, and P2 peptides, MrNV-VLP, and chimeric K5-VLP were used consistently throughout the ELISA experiments. Subsequently, the binding complexes were exposed to 1:2,500 streptavidin-HRP at 37°C for 2 h. Enzymatic products were developed using SureBlue HRP substrate (SeraCare, Milford, MA, USA). The reaction was stopped by rinsing with PBS, and the intensity of the immunoreactive product was recorded using a VersaMax ELISA reader (Molecular Devices, San Jose, CA, USA) at 450 nm. Data are presented as mean ± standard deviation of relative VP28 binding percentages, based on biological triplicates. The inhibition assay was performed similarly to the ELISA experiments mentioned above by including 10 μg of P1 or P2 peptides in 0.08% DMSO or chimeric K5-VLP into the coated tissue extracts and incubating at room temperature for 2 h. Excess inhibitory substances were washed away with PBST and further incubated with 2.5 μg biotinylated VP28. The binding complex was exposed to 1:1,000 anti-His monoclonal antibody at 37°C for 2 h and further exposed to 1:5,000 goat anti-mouse IgG (Abcam) secondary antibody conjugated with hepatocellular protease. The enzymatic product was visualized using a SureBlue HRP substrate (SeraCare). The inhibition percentage was calculated from the mean optical density (O.D.) value obtained from the inhibition groups divided by the mean O.D. value obtained from biotinylated VP28 multiplied by 100.

#### Indirect immunofluorescence microscopy: Labeling of shrimp tissue

For immunohistochemistry on gill tissues, 10 μm-thick cryosections were placed onto coated slides and then rehydrated in PBS-T at room temperature (25°C) for 30 min. Non-specific binding was blocked with 4% BSA in PBS-T for 2 h. Thereafter, a solution of either biotinylated-VP28 protein, biotinylated-P1/P2 peptides, or biotinylated control peptide at the same concentration described in ELISA was added onto hydrated gill sections and incubated at 4°C overnight. Tissues were washed with 0.05% PBS-T, blocked with 4% BSA-PBS at room temperature for 1 h, and incubated with 1:500 Alexa 594-conjugated streptavidin in 4% BSA-PBS at room temperature for 2 h in a dark chamber. After washing, the nuclei were counterstained with 1:5,000 DAPI (4´,6-diamidino-2-phenylindole), and the slides were mounted with ProLong Gold Antifade reagent (Thermo Fisher Scientific, Waltham, MA). Images were acquired using an Olympus FV1000 confocal laser scanning microscope with argon laser lines for emission and excitation filters of 522 ± 5 nm for Alexa 594 and 450 ± 10 nm for DAPI. Additionally, Kalman’s line-by-line scanning mode was employed to minimize fluorescent crosstalk. All images were visualized using the Olympus FluoView software version 4.2a (Olympus, Tokyo, Japan).

#### Indirect immunofluorescence microscopy: Hemocyte labeling

To collect hemocytes, hemolymph was with-drawn from three individual shrimp at the site of the hemosinus, posterior to the carapace, using a 26-G needle equipped on a syringe containing a 10% (w/v) tri-sodium citrate anticoagulant solution. They were further incubated in 24-well plates, each well containing 500 μL of 2× Leibovitz’s L-15 medium supplemented with 15% fetal bovine serum, 0.2% glucose, 0.5% NaCl, and 1% antibiotic/antimycotic mixture (Life Techn-ology, Carlsbad, CA). Attachment of hemocytes was performed at 1 × 10 cells per well at room temperature for 1 h. Thereafter, cells were incubated overnight at 4°C with biotinylated-VP28, -P1/P2, -control peptide, chimeric K5-VLP, or parental MrN-VLP at the same concentrations as those used for ELISA. Cells were fixed with 4% formaldehyde in PBS, extensively washed with PBS, and then blocked with 10% fetal bovine serum in PBS at room temperature for 1 h. They were exposed to 1:500 Alexa 594-conjugated streptavidin (Thermo Fisher, cat# S11227) in 4% BSA in PBS at room temperature for 2 h in a dark chamber. After several washes with PBS, the cells were counterstained with 1:3,000 DAPI and mounted with ProLong Gold Antifade reagent (Thermo Fisher Scientific). The mounted slides were observed using an Olympus FV1000 confocal laser scanning microscope under the aforementioned conditions.

### Statistical analysis

The calculation of shrimp sample size used in this study was calculated using the G*Power 3.1 program (Informer Technologies Inc., Raleigh, NC, USA) with 95% confidence level. All collected data were expressed as mean ± standard error of triplicate biological analyses. Statistical significance was analyzed by one-way analysis of variance followed by Dunnett’s *post hoc* test using GraphPad Prism v.10.1.1 (GraphPad Software Inc., Boston, MA, USA). Confidence levels of p < 0.05 and p < 0.01 were used for all statistical analyses.

## RESULTS

### Expression of rVP28 and K5-VLP validation

**Figure 1:**
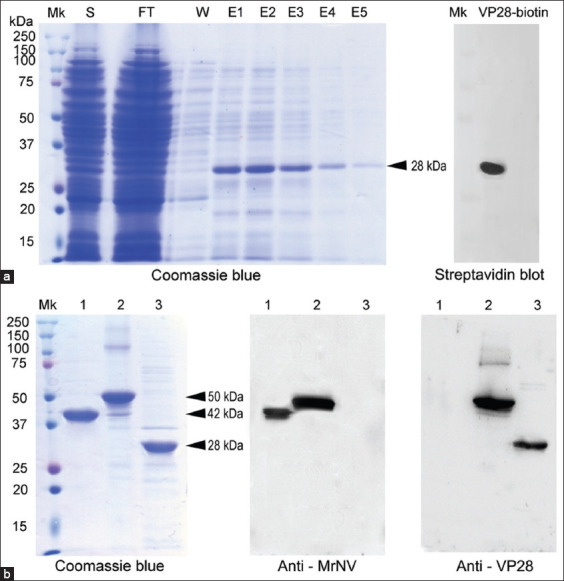
Expression and purification of (a) recombinant (r) VP28 and (b) chimeric MrNV capsid protein. Protein profiling by sodium dodecyl sulfate-polyacrylamide gel electrophoresis and Coomassie blue staining is in the left panel, and Western blotting is in the right panel. VP28=Viral protein 28, MrNV=*Macrobrachium rosenbergii* nodavirus.

Recombinant VP28 protein was successfully expressed in *E. coli* (Rosetta), yielding a highly purified (>95%) single band at approximately 28 kDa, as demonstrated by Coomassie blue staining and Western blotting (Figure 1a). Biotinylation of the VP28 protein was confirmed through its reactivity with streptavidin-HRP after transfer to a PVDF membrane (Figure 1a, right panel), supporting its suitability for ELISA and in-tissue binding experiments. For the chimeric K5-VLP, the purified capsid protein displayed an upward molecular mass shift to 50 kDa (Figure 1b, lane 2), consistent with the incorporation of the VP28 peptide, compared with the parental MrNV-VLP capsid protein at 42 kDa (Figure 1b, lane 1). This observation aligned with the predicted molecular mass derived from the complete amino acid sequence of K5-VLP, which estimated the expressed protein at approximately 50 kDa. In this context, Western blotting with anti-MrNV and anti-VP28 antibodies confirmed the successful incorporation of both protein domains into the chimeric K5-VLP construct (Figure 1b, right panel).

### Presentation of the P1 peptide on the VLP

**Figure 2:**
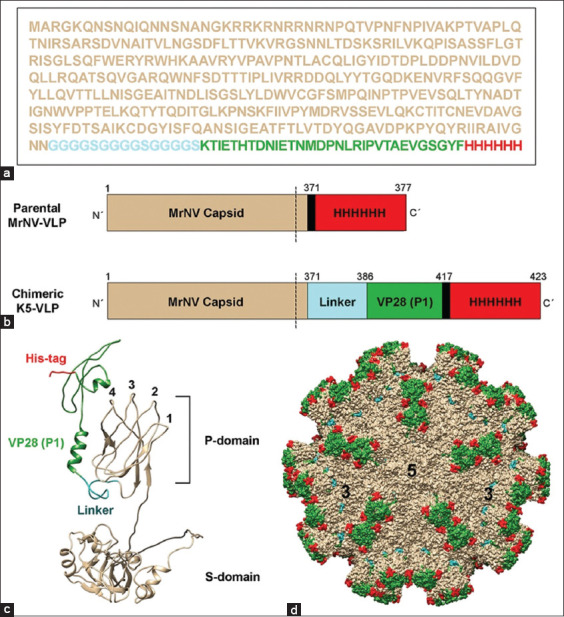
Deduced structures of the two- and three-dimensional monomeric and multimeric chimeric K5-VLP models. (a) Full amino acid sequence and (b) linear alignment of chimeric K5-VLP demonstrated an extension of VP28_(35-65)_ or P1 peptide (green) to the C-terminal domain of MrNV protein by a (GGGGS)_3_ linker (cyan). Three dimensional models of (c) single subunit, and (d) multimeric VLP in space filling model. Red=Hexahistidine tag. K5-VLP=Virus-like particle, MrNV=*Macrobrachium rosenbergii* nodavirus, VP28=Viral protein 28.

Structural annotation of both linear and three-dimensional models, including monomeric and multimeric assemblies, was performed. Figure 2 illustrates the genetic fusion of the P1 peptide to the MrNV capsid protein via a flexible linker (Figures 2a and b). The P1 peptide extends from the protruding (P) domain near the fourth pillar of the individual capsid subunit (Figure 2c). Three-dimensional projections of the assembled chimeric K5-VLP confirmed the surface exposure of the P1 peptide (green) on the protruding domain of the capsid (Figure 2d).

### Icosahedral orientation of the chimeric K5-VLP

**Figure 3:**
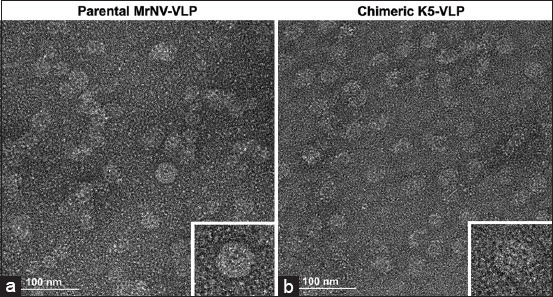
Electron micrographs illustrating (a) Parental MrNV-VLP and (b) chimeric K5-VLP that form an icosa- hedral mulberry-like structure. Bars=100 nm. K5-VLP=Virus-like particle, MrNV=*Macrobrachium rosenbergii* nodavirus.

TEM was employed to determine whether the expressed chimeric K5 capsid proteins self-assembled into icosahedral VLPs (K5-VLP). TEM images at 50,000× magnification confirmed that K5-VLP formed typical icosahedral, mulberry-like particles (Figure 3b), closely resembling the parental MrNV-VLP (Figure 3a), previously shown to form icosahedral particles with diameters of 26–27 nm. These findings suggest that the addition of the P1 peptide near the fourth pillar of the P-domain does not disrupt the assembly of VLPs.

### Tissue binding profiles of VP28, P1, and P2 peptides

**Figure 4:**
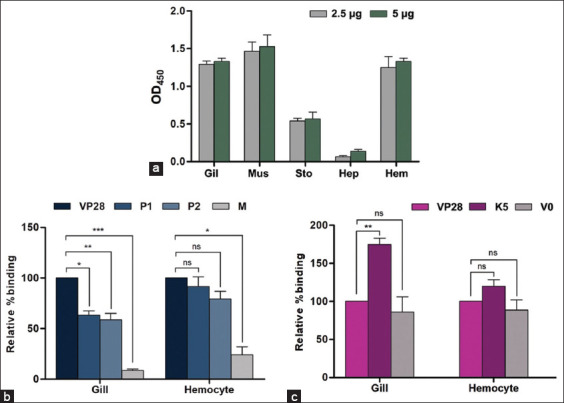
Binding of (a) rVP28 in various shrimp tissues and its comparative binding .with (b) its derivative peptides (P1 and P2), and (c) K5-VLP in gill and hemocyte. All bars were calculated from triplicated experiments and expressed as mean ± standard deviation. K5=Chimeric K5 virus-like particle, V0=MrN-VLP, VP28=Viral protein 28, Gil=Gill, Mus=Muscle, Sto=Stomach, Hep=Hepatopancreas, Hem=Hemocyte.

Recombinant VP28 is known to induce immune responses in shrimp tissues [20]. However, the direct binding profiles of VP28 and its multimeric form on VLPs have not been previously quantified across shrimp tissues using both ELISA and immunofluorescence. Accordingly, we first examined the binding of rVP28 to five shrimp tissue extracts: Gill, muscle, stomach, hepatopancreas, and hemocytes. VP28 showed strong binding to several tissue extracts at both 2.5 μg and 5 μg. The highest binding signals, ranging from 1.20 to 1.25 O.D., were observed in gills (1.2), muscles (1.25), and hemocytes (1.25), while moderate binding was recorded in the stomach at 0.48 O.D. (Figure 4a). The lowest binding level was observed in the hepatopancreas (O.D. = 0.09). Subsequently, the binding efficiency of rVP28 was compared with its derivative peptides, P1 and P2, and chimeric K5-VLP, in gill and hemocyte tissues–identified as the principal sites of infection and propagation [21]. At a concentration of 0.64 nmol, P1 and P2 peptides exhibited relative binding efficiencies of 63.40% and 58.64%, respectively, compared to full-length VP28 in gill tissue extracts (Figure 4b, left panel). In hemocytes, the binding levels of P1 and P2 were 91.68% and 70.26%, respectively (Figure 4b, right panel). The control mock (M) peptide exhibited much lower binding, with only 8.38% in gills and 24.05% in hemocytes relative to VP28 (Figure 4b).

### Tissue-binding profiles of K5-VLP

Chimeric K5-VLP demonstrated binding levels of 174.6% relative to VP28 in gill lysates and 120.0% in hemocyte lysates (Figure 4c, K5 bars), outperforming the single-subunit VP28 peptide. Notably, the parental MrNV-VLP (V0) exhibited relative binding levels of 85.98% in gill lysates and 88.37% in hemocyte lysates–slightly lower than that of VP28 (Figure 4c). This relatively strong binding by parental MrNV-VLP was anticipated, as MrNV is also known to interact with unidentified receptors in shrimp tissues [22].

### Inhibition of the binding of VP28

**Figure 5:**
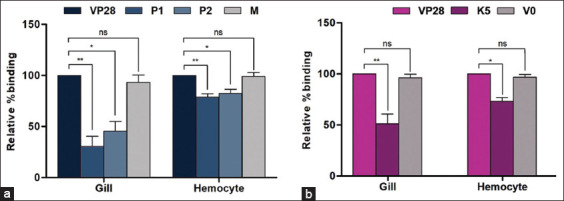
Inhibition assay of rVP28 binding to (a) P1/P2 peptides or (b) chimeric K5-VLP in gill and hemocyte tissues. The data were expressed as the relative percentage of binding with rVP28. Asterisks (*, **) represent significant differences at p < 0.5 and p < 0.01, respectively. V0=Parental MrNV-VLP, K5=Chimeric K5-VLP. K5-VLP=Virus-like particle, VP28=Viral protein 28.

Inhibition assays using P1 and P2 peptides revealed their capacity to competitively reduce VP28 binding by 70% and 55%, respectively, in gill tissues (Figure 5a, left panel). In hemocytes, P1 and P2 achieved only 20% and 18% inhibition, respectively (Figure 5a, right panel). The control peptide showed only 10% inhibition of VP28 binding. Chimeric K5-VLP inhibited VP28 binding by 50% in gill tissues (Figure 5b, dark-pink bar), consistent with trends observed for the peptide inhibitors. In hemocytes, only 27% inhibition was observed (Figure 5b). The inhibitory effect of the parental MrNV-VLP was limited to 4%–5% in both gill and hemocytes (Figure 5b, gray bar). Together with the binding results from Figure 4, these findings suggest that WSSV and MrNV may not share the same receptors but instead utilize distinct receptor sets on host cell surfaces.

### Microscopy validation of VP28 and K5-VLP binding and inhibition assays

**Figure 6:**
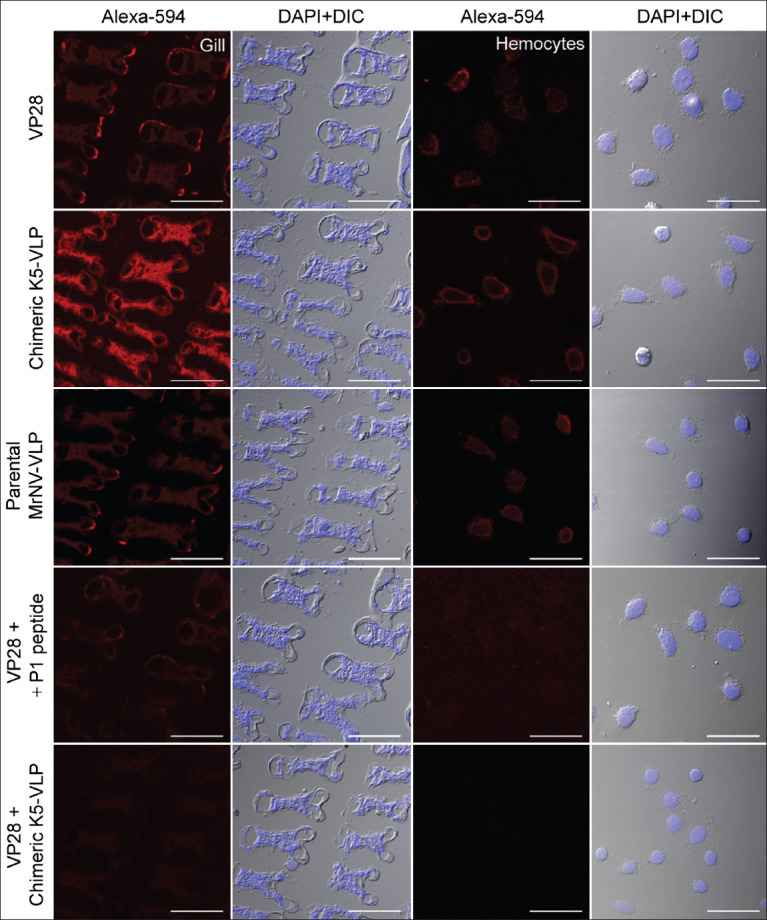
Confocal fluorescence micrographs of VP28 binding and inhibition by P1/P2 or chimeric K5-VLP in gill tissue and hemocytes. The red fluorescent signals (first column) represent bindings of VP28 (first row), chimeric K5-VLP (second row), and parental MrNV-VLP (third row). Inhibition of VP28 with P1/P2 peptides and chimeric K5-VLP were in the fourth and fifth rows, respectively. Blue fluorescence represents nuclear counterstaining with DAPI. Bars=50 μm, VP28=Viral protein 28, K5-VLP=Virus-like particle, MrNV=*Macrobrachium rosenbergii* nodavirus.

Immunohistochemical staining of shrimp tissues showed strong red fluorescence upon treatment with biotinylated VP28 in all gill tissue cell types (Figure 6, first row). Notably, intense fluorescent signals were also observed in gill tissues treated with chimeric K5-VLP and parental MrNV-VLP (Figure 6, second and third rows), consistent with the ELISA results. A similar fluorescence pattern, encircling the cell periphery, was observed for both K5-VLP and MrNV-VLP (Figure 6, rows 1–3). In inhibition assays, co-treatment with P1/P2 peptides and chimeric K5-VLP led to a visible reduction in fluorescence intensity in both gill tissues and hemocytes (Figure 6). Inhibition by chimeric K5-VLP consistently showed a greater reduction in VP28 binding compared to peptide-based inhibition, across both gill tissues and hemocytes (Figure 6, fifth row). Altogether, this study establishes a structural-functional relationship between VLP-displayed VP28 epitopes and their interaction with host tissues.

## DISCUSSION

### Tissue tropism and binding specificity of VP28 and its derivatives

The VP28 of WSSV, particularly its extracellular domain (residues 35–95), plays a crucial role in facilitating viral entry into shrimp tissues and stimulating the shrimp immune system [14]. In this study, we further demonstrated that the P1 and P2 peptides of VP28 effectively bound to multiple shrimp tissues, including gills, muscles, stomach, and hemocytes, while showing negligible binding to the hepatopancreas–similar to findings reported by Chang [23]. The widespread tissue-binding pattern of VP28 may reflect WSSV’s initial tropism for gill and hemocyte tissues, facilitating systemic dissemination via hemocytes through the shrimp’s open circulatory system [2, 5, 10, 21]. The lack of VP28 binding and WSSV infection in the hepatopancreas remains poorly understood and warrants further investigation, despite the organ’s high vascularization and its role in digestion, similar to the stomach-which does show notable VP28 interaction (Figure 4). The detection of VP28 in gill and stomach tissues corroborates earlier studies identifying these organs as primary replication sites for WSSV [21]. In addition, the observed binding of VP28 to hemocytes aligns with previous findings that suggest VP28 interacts with unidentified receptors on the hemocyte surface, triggering phagocytic activity [11].

### Comparative binding efficiency of full-length VP28, peptides, and K5-VLP

Comparative binding analysis between the full-length VP28, its derivative peptides P1/P2, and the chimeric K5-VLP showed that the short peptides exhibited lower binding affinity than full-length VP28. Notably, K5-VLP demonstrated a 1.7-fold higher binding efficiency than full-length VP28 (Figure 4). These findings suggest that VP28’s binding affinity is likely conformation dependent, requiring an intact tertiary structure for optimal receptor interaction and effective host-virus engagement–similar to many other viral proteins that interact with host cells [24, 25]. Therefore, fragmented VP28 peptides may lack the necessary structural integrity to achieve effective ligand–receptor stoichiometry.

### Conformational advantages of multimeric VP28 display

The tetrameric form of VP28 has been shown to exhibit improved host cell interaction and greater interference with WSSV during viral challenge studies [15, 16]. This aligns with our findings that multimeric VP28 peptides displayed on VLPs exhibit enhanced binding efficiency to shrimp tissues compared to monomeric VP28 (Figure 4c). Displaying VP28 on the protruding C-domain of MrNV-VLP promotes surface exposure and mimics the native viral architecture on the external surface of the capsid [22], enhancing viral-like conformation and facilitating host cell interaction. In this context, we previously demonstrated by Grataitong *et al*. [26] that decorating the MrNV-VLP with a cancer-targeting peptide (named GE11 peptide which has been screened through phage display and specific to epidermal growth factor receptor) enhanced its specific interaction with a colorectal cancer cell line, whereas the unmodified MrNV-VLP showed negligible interaction.

### Intracellular trafficking of WSSV and the role of VP28

WSSV infection can be effectively blocked by disrupting the molecular pathways the virus uses for host cell invasion. In addition to the virus-host surface interactions mentioned above, intracellular trafficking of WSSV within the host cytoplasm depends on the endosomal sorting complex required for transport (ESCRT) machinery, which facilitates endosomal translocation via interactions between viral envelope proteins (e.g., VP28) and host endosomal proteins such as Tsg101 and Rab7 [27]. Notably, silencing the Rab7 gene has been shown to significantly inhibit WSSV replication by disrupting endosomal trafficking [27], likely resulting in virion mistargeting to lysosomes, where they are degraded by hydrolytic enzymes [28]. After escaping the endosome, other envelope proteins, such as VP664 and VP15, mediate the nuclear translocation of WSSV by engaging with nuclear localization signals and importins, allowing viral replication to proceed within the host nucleus [27, 29, 30]. Alternatively, VP15 has been reported to bind the gC1qR receptor–a complement system component–potentially activa-ting the innate immune response against WSSV in *Marsupenaeus* japonicus [31].

### Immunostimulatory potential of MrNV-VLP and Its chimeric forms

Our previous studies by Chen *et al*. [32], Jariyapong *et al*. [33] and Wen *et al*. [34] have shown that MrNV-VLP administration upregulates shrimp immune-related genes, including caspase, crustin, astakine, heat shock protein 90, prophenoloxidase-activating enzyme, and defender against apoptotic death 1, thereby enhancing the host’s antiviral defense.

### Multifunctional role of K5-VLP as an antiviral platform

Leveraging this body of knowledge, chimeric VLPs can be designed as multi-functional antiviral platforms. In the case of WSSV, the surface-expressed VP28 on K5-VLP acts as a first barrier by competitively interfering with native virion binding. Once internalized, K5-VLP may disrupt virus-ESCRT interactions, potentially preventing viral trafficking to the endosome. In addition, the MrNV-VLP core can act as an innate immune stimulant, enhancing the host’s antiviral response. Furthermore, chimeric VLPs offer an added advantage by encapsulating nucleotide-based therapeutic agents, such as double-stranded RNA (dsRNA)–one of the most effective compounds for halting viral replication in host cells [35]. Collectively, the chimeric K5-VLP and similar surface-engineered MrNV-VLPs present a potent, multi-targeted strategy for combating viral infections in shrimp aquaculture.

## CONCLUSION

In this study, we successfully engineered a chimeric VLP (K5-VLP) presenting a multimeric display of the WSSV envelope-derived VP28 peptide on the protruding domain of the MrNV capsid. The chimeric K5-VLP demonstrated superior binding affinity to shrimp gill and hemocyte tissues-showing 1.7-fold higher binding efficiency compared to the full-length recombinant VP28, and significantly outperforming its P1 and P2 derivative peptides. These findings confirm the importance of multivalent and conformation-preserved presenta-tion of VP28 in enhancing tissue-specific interaction. Furthermore, the K5-VLP effectively competed with native VP28 in inhibition assays, reducing its binding to shrimp tissues by up to 50%, indicating strong viral interference potential. Immunohistochemical analysis also revealed the strong localization of K5-VLP in target tissues, validating its biological activity and functional surface exposure.

Practical implications of this work include the potential deployment of K5-VLP as a next-generation antiviral agent or vaccine platform in shrimp aqua-culture. Its ability to both mimic native viral structure and induce immune-relevant tissue targeting offers a dual mechanism: competitive viral blocking and host immune priming. In addition, the structural compatibility of MrNV-VLP with peptide fusion and its capacity for encapsulating antiviral cargo, such as dsRNA, supports its application as a modular delivery vehicle.

The strengths of the study lie in its integrated structural-functional validation of the chimeric VLP, the use of multiple binding and inhibition assays, and its demonstration of immunofluorescence-based localization, providing a thorough *in vitro* assessment of the antiviral potential. The inclusion of both full-length and truncated VP28 forms enabled a comparative analysis, highlighting the conformational dependence of effective receptor engagement.

Limitations of this study include the lack of *in vivo* viral challenge trials to directly evaluate the protective efficacy of K5-VLP under natural infection conditions. Furthermore, the specific host receptors mediating VP28 interaction remain unidentified, limiting mechanistic insights into host-pathogen dynamics.

Future scope includes *in vivo* challenge trials to evaluate the protection efficacy against WSSV, optimization of the VP28 fusion design to enhance immunogenicity, and encapsulation of therapeutic nucleotides (e.g., dsRNA) within the VLP core to achieve multifunctional delivery. Molecular studies identifying the exact host receptors of VP28 would also provide critical insight into host-pathogen co-evolution and enable targeted antiviral strategies.

In conclusion, the chimeric K5-VLP represents a promising multifunctional antiviral candidate that integrates structural mimicry, viral interference, and immunostimulatory capacity. Its modular design offers a scalable and customizable approach for combating viral diseases in shrimp aquaculture, contributing to more sustainable and disease-resilient farming practices.

## DATA AVAILABILITY

The supplementary data can be available from the corresponding author upon a request.

## AUTHORS’ CONTRIBUTIONS

SJ: Conceptualization, methodology, formal analysis, writing - original draft. PJ: Conceptualization, methodology, validation, writing - review and editing, funding acquisition. OT: Methodology, formal analysis. SB: Methodology, formal analysis. CC: Supervision. MS: Supervision. SA: Supervision. AW: Investigation, supervision, validation. WW: Conceptualization, supervision, and writing - review and editing. All authors have read and approved the final manuscript.
